# Diamonds in the not-so-rough: Wild relative diversity hidden in crop genomes

**DOI:** 10.1371/journal.pbio.3002235

**Published:** 2023-07-13

**Authors:** Sherry Flint-Garcia, Mitchell J. Feldmann, Hannes Dempewolf, Peter L. Morrell, Jeffrey Ross-Ibarra

**Affiliations:** 1 Plant Genetics Research Unit, United States Department of Agriculture, Agricultural Research Service, Columbia, Missouri, United States of America; 2 Department of Plant Sciences, University of California, Davis, California, United States of America; 3 The Global Crop Diversity Trust, Bonn, Germany; 4 Department of Agronomy and Plant Genetics, University of Minnesota, Minneapolis, Minnesota, United States of America; 5 Department of Evolution and Ecology, Center for Population Biology, and Genome Center, University of California, Davis, California, United States of America; University of California, Davis, UNITED STATES

## Abstract

Crop production is becoming an increasing challenge as the global population grows and the climate changes. Modern cultivated crop species are selected for productivity under optimal growth environments and have often lost genetic variants that could allow them to adapt to diverse, and now rapidly changing, environments. These genetic variants are often present in their closest wild relatives, but so are less desirable traits. How to preserve and effectively utilize the rich genetic resources that crop wild relatives offer while avoiding detrimental variants and maladaptive genetic contributions is a central challenge for ongoing crop improvement. This Essay explores this challenge and potential paths that could lead to a solution.

## Abstract 2

The wild relatives of domesticated plants harbor tremendous genetic diversity that has the potential to help adapt crops to changing climates and future challenges. This Essay looks at historical admixture between crops and their wild relatives, and how these traditional varieties provide an evolutionary filter that has selected for useful diversity.

## Introduction

Plant domestication is a process that began with cultures around the world experimenting with alternative means of food production. These experiments have expanded, undoubtedly, beyond the imaginations of their originators. Much of the planet’s surface is now involved, and most of human sustenance derives from experiments that originated among fewer than 1,000,000 humans starting 10 to 12,000 years before present. Domestication has involved a mix of intentional (or “artificial”) selection and unintentional (sometimes “unconscious”) selection—the simple result of differential survival and reproduction among individuals in a novel environment [1].

**Fig 1:**
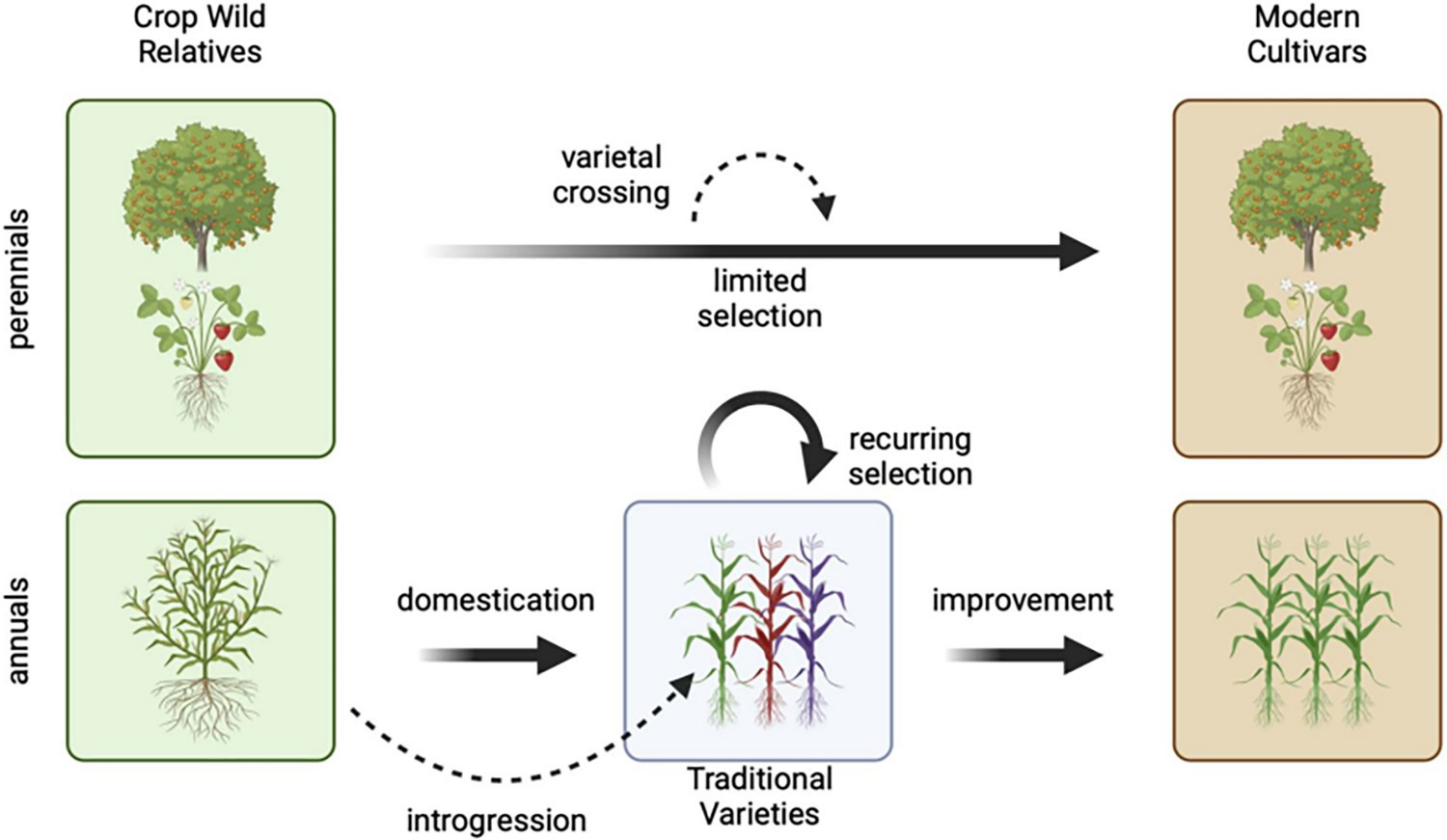
From crop wild relatives to modern cultivars. Modern cultivars of perennial crops like strawberry and citrus have often undergone relatively few generations of selection from a common ancestor with their wild relatives compared with many annual cereal crops, and modern cultivars of many perennial species may have resulted from hybridization among wild taxa or earlier varieties. For annual crops like maize, domestication involved an extended process of hundreds or thousands of generations of selection resulting in traditional varieties. Traditional varieties regularly exchange genes with crop wild relatives and are shaped by continual selection imposed by farmers and adaptation to their environment. Adaptation to modern agricultural conditions, here identified as crop improvement, is a relatively recent process usually involving only tens of generations of selection. Figure was created using BioRender.com.

Changes resulting from domestication occurred slowly. Archeological evidence demonstrates protracted, gradual change that lasted thousands of generations for many domesticates (Box 1) [2]. Evolution continues today in traditional populations outside of formal breeding programs [3]. While there are clear examples of genetic loci that have a major impact on domestication phenotypes, in each cultivated species the process likely involved changes in hundreds or thousands of genes [4–7]. This results in a continuum of morphological and genetic differentiation, but crops and their relatives can nonetheless be usefully categorized into 3 broad groups (Fig 1). The first are extant populations of wild plants that share a common (wild) ancestor with domesticates some time in the past. These extant populations are not the direct progenitors of crops but can be identified as crop wild relatives. They include taxa most closely related to a domesticate, as well as more distantly related taxa, especially those that can hybridize with the domesticate. Second are domesticated plants that result from intentional and unintentional selection by indigenous peoples, known as traditional varieties (often called landraces). These are often diverse and continue to be cultivated and selected in smaller-scale agricultural settings worldwide. Third are modern cultivars, which have been developed in the past century from directed breeding efforts following the advent of industrial agriculture. Modern cultivars are typically highly adapted to current agronomic environments and display desired characteristics often absent from crop wild relatives and traditional varieties, such as high, stable yields or ease of processing and transportation.

**Box 1. Glossary**

**Domesticates**

Plants that have coevolved with humans. Most domesticates rely on humans for survival and reproduction.

**Deleterious alleles**

Alleles that decrease the survival or reproductive capacity of an organism.

**Genetic drift**

One of the fundamental evolutionary processes, genetic drift refers to stochastic changes in allele frequencies unrelated to an allele’s impact on fitness.

**Purifying selection**

Natural selection that removes deleterious alleles from a population.

**Germplasm**

Genetic resources related to the species being studied, including wild relatives, unimproved populations such as landraces or heirlooms, and improved varieties. These genetic resources—usually seeds but possibly including living plants or tissue culture—are collected and maintained for long-term preservation and are commonly used in genetic studies or breeding programs.

**Introgression**

Introduction of genetic material from one taxa or species into another. Introgression may occur naturally via hybridization or via inbreeding by traditional crossing and repeated backcrossing to a recurrent parent. The size of the introgressed region depends on the local recombination rate and how many backcrosses have occurred; each backcross with the recurrent parent results in reduction of the donor genome by approximately 50%.

**Elite recurrent parent**

The recipient parent of an introgression, typically an improved variety with high yield or superior quality, that is otherwise lacking a particular trait to be introduced from a donor variety.

**Heterotic patterns**

In species that exhibit heterosis or hybrid vigor, there are often specific germplasm combinations that result in higher or lower levels of heterosis. Low levels of heterosis may result when crossing individuals from the same heterotic group, while crosses between individuals from 2 different heterotic groups result in higher levels of heterosis.

The evolutionary path from wild species to domesticate is different for each crop (Fig 1); thus, the demographic and genetic history of each species is unique. Annual species typically experienced greater reductions in diversity and more generations of strong selection than perennial crops; perennials often retain more diversity but also more deleterious alleles (Box 1) or genetic load [8,9]. Long-lived perennial and clonally propagated species may have undergone fewer generations of differentiation from their wild progenitors, and modern cultivars may thus differ little from traditional varieties. For example, only a handful of generations and a few genetic crosses separate citrus or strawberry modern cultivars from their wild progenitors [6,10]. Differences in plant mating systems likely affected opportunities for gene flow, and the structuring of genetic diversity across populations [11] and preexisting ecological relationships may have preadapted some species to more rapid domestication [12]. In addition to these biological factors, historical contingencies may have played a significant role in the evolution of many crops [13], including whether domestication happened once, as in maize [14], or multiple times, as in barley [15] and amaranth [16].

**Fig 2:**
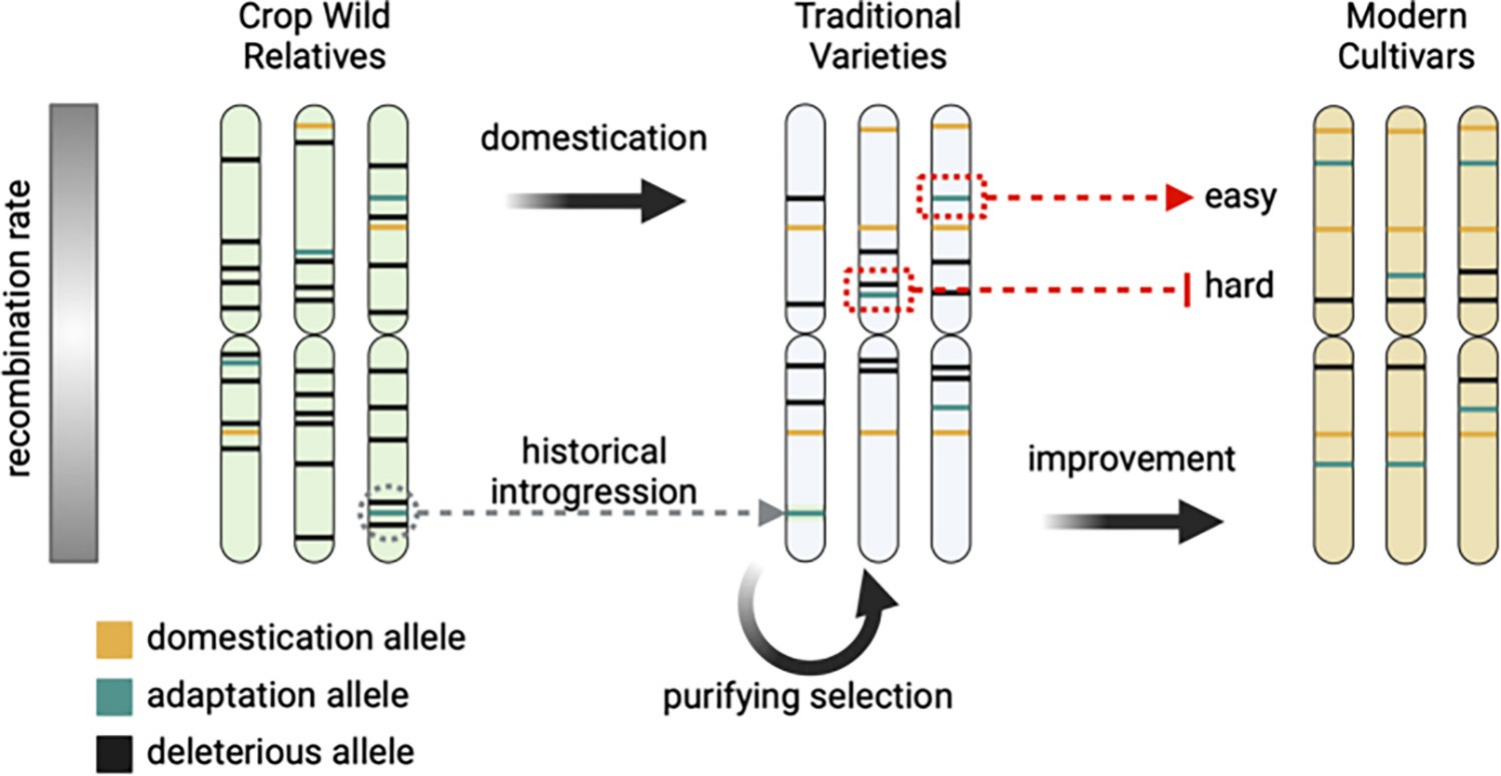
Deleterious load and crop evolution. Shown are 3 chromosomes sampled from populations of crop wild relatives, traditional varieties, and modern cultivars. Selection during crop evolution increases the frequency of domestication-related alleles, which are beneficial in agronomic settings, but not necessarily alleles for adaptation, which may only be beneficial in specific environments. Deleterious alleles are often concentrated in low recombination regions of the genome (white on the recombination scale bar) and preferentially removed by purifying selection, but some are fixed during the process of domestication and improvement. Adaptive alleles linked to deleterious alleles are difficult to introgress because of their negative impacts on fitness or agronomic traits (red dashed line labeled “hard”), but adaptive alleles far from deleterious alleles can be easily introgressed (red dashed arrow labeled “easy”). But the long-term combined action of introgression, recombination, and selection has allowed the historical introgression of “hard” adaptation alleles from crop wild relatives into traditional varieties (gray dashed arrow), where they could then be more easily incorporated into modern cultivars. Figure was created using BioRender.com.

Whether domestication entailed thousands of generations of gradual selection or the extraction of a single clonal genotype from wild populations, it nearly always results in the loss of genetic diversity in traditional varieties and modern cultivars compared with crop wild relatives. An understanding of the extent of loss in genetic diversity in domesticates is relatively new [17], as earlier natural history often emphasized the diversity of phenotypic forms in cultivated varieties [18,19]. But molecular markers reveal that crop diversity largely represents a subset of that in wild relatives [20], and a loss of diversity is also evident in comparisons of the genetic variation underlying agronomic phenotypes [21]. The initial stages of domestication almost invariably involved only a subset of crop wild relative individuals, and much of the loss of diversity likely resulted from this sampling process and genetic drift (Box 1) [22]. But allelic diversity is also lost by positive selection fixing alleles relevant for domestication and purifying selection (Box 1) removing deleterious alleles (Fig 2). Modern breeding accentuates both drift and selection, resulting in an ever-narrowing base of diversity available for further improvement [23,24]. Indeed, while perennial crops diverge from their wild relatives by fewer generations and thus may better capture their wild relative genotypes, diversity in perennial modern cultivars is often low because only a small number of varieties are in widespread use [25,26].

In this Essay, we argue that allelic diversity from crop wild relatives likely already exists in cultivated populations conserved in germplasm (Box 1) repositories. These alleles have been tested by evolution in an agronomic background. Many are of sufficient age that the 2-fold sieve of recombination and selection have separated them from linked deleterious variants. Surveying domesticated traditional varieties for functionally relevant variation from crop wild relatives may thus greatly facilitate the identification and incorporation of useful wild diversity into modern breeding programs.

### Using crop wild relatives directly in breeding programs

Crop wild relatives contain a wealth of alleles that were lost during domestication and improvement [27–29]. These alleles can be valuable sources of desirable traits such as disease and insect resistance, abiotic stress resistance, flavor and nutritional quality, and plant growth and health. Incorporating this diversity can improve breeding populations, support emerging markets and novel products, and contribute to adapting crops to changing climates [29–31]. For any given crop, there are often multiple crop wild relatives that could potentially be useful and may vary in terms of genetic distance, interfertility, and maintenance of useful traits relative to the relevant modern cultivar. Prioritizing which samples and populations are maintained in collections, evaluated for desirable traits, and incorporated into modern cultivars is both necessary and challenging.

### Crop wild relatives and traditional varieties are a good source of novel variation

Crop wild relatives and traditional varieties share much of their genetic makeup with modern cultivars. In the initial stages of cultivation and in incipient domesticates, long segments of the genome will be shared with wild relatives. These relationships reflect identity by descent (IBD) from parents to progeny. The size of IBD regions is reduced each generation by recombination and is dependent on factors such as the outcrossing rate and diversity within populations. Individuals from closely related populations can share large regions of IBD over hundreds of generations [32]. Sharing of large regions of IBD is also a hallmark of recent introgression (Box 1) and distinguishing between shared ancestry and recent introgression can be difficult.

Because domestication is a recent evolutionary process, the majority of gene-level variants in modern cultivars, including single-nucleotide changes and insertions and deletions, are a subset of those found in crop wild relatives (c.f. [33,34]). Nonetheless, modern cultivars have also diverged as a result of genetic drift, selection, and the accumulation of new mutations. These factors are particularly important in clonally propagated species where recombination is largely absent [8]. Variants are also arranged into new haplotypes by recombination. This can include multiple combinations of functional variants such as amino acid changing mutations and regulatory elements.

Direct introgression of crop wild relative alleles is a major strategy for increasing genetic diversity and genetic variation in commercial breeding programs. Breeders typically use introgression to respond to an emerging threat or existing deficiency in the commercial germplasm collection. Genetic variation is required for breeders to make genetic gains, and alleles that can confer a selective advantage for emerging threats such as a new disease or climatic extremes may not exist in modern cultivars. In this case, researchers and breeders look for the desired trait variation in crop wild relatives in hopes of finding individuals that contain alleles with large genetic effects capable of producing adapted progeny. This approach has been successful in many cases [35], including for disease resistance [36,37] and abiotic stress [38]. Even with genes of large effect for domestication or improvement where beneficial alleles have been fixed in modern cultivars, agronomically relevant variation may exist in crop wild relatives. For example, branching was selected against during sunflower domestication but was later reintroduced from a wild relative to facilitate hybrid breeding [39]. Selection at domestication or improvement loci often results in the fixation of one or a few haplotypes, sometimes inadvertently fixing inferior alleles at nearby linked genes [40,41]. Even at loci directly targeted by selection during breeding, crop wild relatives often exhibit diverse allelic series [42,43], including variation at loci conferring favorable variation for yield components [44,45].

Incorporating novel diversity from crop wild relatives into commercial breeding populations is the fundamental goal of “pre-breeding” for many crops. The pre-breeding process requires phenotyping crop wild relatives, identifying key lineages (donors) with beneficial or novel alleles, and introgressing the donor alleles. Introgression is most commonly done by crossing diverse germplasm with the relevant modern cultivar in conjunction with selection for the novel trait or allele using phenotype, genetic markers, or predicted breeding values [46]. One successful example of pre-breeding is the Germplasm Enhancement of Maize, a coordinated effort of the US Department of Agriculture, university breeders, and industry partners to widen the germplasm base of commercial hybrid corn in the United States through the incorporation of traditional variety alleles in elite modern cultivars [47]. Some sectors of private industry also invest in efforts such as “discovery breeding,” where broader germplasm, including traditional varieties and crop wild relatives, are explored to improve modern cultivars.

### Crop wild relatives are replete with maladaptive alleles

By definition, crop wild relatives are unimproved or less improved than modern cultivars and hence harbor alleles that are maladaptive under modern agronomic practices [48]. These maladaptations include photoperiod sensitivity, plant architectures less amenable to harvest, or susceptibility to biotic and abiotic stresses, all of which can dramatically affect yield and product quality (e.g., [29,49]). In addition, crop wild relatives are often not suited for the long-term storage or long-distance transportation systems of modern food supply chains. For these reasons, they are usually not used directly in breeding programs and are instead subjected to the pre-breeding process described above. Following hybridization of the crop wild relative donor with an elite recurrent parent (Box 1), each backcross with the recurrent parent results in a loss of half of the existing donor genome. For example, after 5 generations of backcrossing, the expected proportion of the donor parent is only 3.125%. Recombination during this process is relatively limited, however, resulting in the introgression of the large chromosomal regions surrounding a target locus. When selecting for a single locus, backcrossing will also often result in the introgression of off-target regions elsewhere in the genome. This contributes to a form of linked selection [50] known as linkage drag and frequently results in a decrease in agronomic performance as genes that are not the direct targets of selection tend to carry alleles that are detrimental in a modern cultivar background. Genome-scale genetic data have revealed evidence of linkage drag in many crops [51,52], and recent analyses in sunflower showed not only that linkage drag results in decreasing yield, but also that introgressions from more distantly related species are more deleterious than those from closely related taxa [53].

One solution to linkage drag is marker-assisted backcrossing, where flanking markers are used to track the desired allele in a breeding population and to make selections, and genome-wide markers are used to actively select against the remaining donor genome and for the recurrent parent, effectively prioritizing recombination events close to the target locus. The added expense and effort of implementing marker-assisted backcrossing in a breeding program is such that it essentially requires an allele with a large effect to recover the value. In addition, the result is unlikely to be a single gene introgression; larger introgressions can contain dozens to hundreds of genes depending on the genomic context. Each desirable allele will reside in a region of the genome that may contain numerous maladapted alleles, and the combination of recombination rate and haplotype structure (whether beneficial and deleterious alleles are on the same or different haplotypes) will determine the likelihood of breaking up linkage blocks (Fig 2). In the end, the merits and consequences of introgression will depend on several factors, including variation present in the breeding program and the number and effect size of the loci underlying the trait to be introgressed [54].

Overall, while the long-term advantages of increasing diversity and adding functional variation from crop wild relatives are well understood, the short-term challenges are often sufficient to prevent the effective utilization of such wild relatives in breeding programs. This is especially true in industry settings focused on short-term profits (though there are some notable exceptions). The commitment to existing heterotic patterns (Box 1) makes wide crosses with wild relatives even less palatable for hybrid crops. The combination of linkage drag, logistical challenges with backcrossing and marker-assisted selection, and the time scale involved (many years) make the effort required to introgress crop wild relative alleles often not worth the gain. Exceptions to this tend to be large-effect loci where significant agronomic gains are clear [29].

### Crop wild relative alleles have actively introgressed into traditional variety germplasm

Historically, domestication has often been portrayed as the split between cultivated plants and their crop wild relatives. However, empirical studies from a variety of systems highlight that domestication was a complex process that unfolded across a diverse landscape and involved genetic exchange both with a crop’s direct progenitor, as well as with additional wild relatives [55,56]. Human dissemination of crops from centers of origin often happened relatively quickly; crops in the Fertile Crescent, for example, are estimated to have spread from their center of origin at a rate of 1 km/yr [57] and maize spread from the lowlands of Mexico to the Andes in South America in less than 3,000 years [58]. This rapid diffusion forced crops to quickly adapt to new growing environments but also provided the opportunity for hybridization with locally adapted wild relatives. For example, in wheat and other complex polyploid plants, hybridization with wild relatives was essential to the formation of modern cultivated forms [59]. In scarlet runner bean, a complex history of introgression from wild relatives spans both ancient and recent crop evolution [60]. And in numerous crops such as avocado [61], citrus [62], and apple [63], modern varieties are the result of complex patterns of introgression from one or more wild relatives. Indeed, evidence suggests the vast majority of food crops actively hybridize with wild relatives in some part of their range [64].

Far from being accidental or detrimental, gene flow with crop wild relatives has often been instrumental in the evolution of domesticated taxa. In maize, for example, a meaningful subset of recent selection in traditional varieties has been for alleles introgressed from a wild relative [65], and introgression from a different wild relative contributed to highland adaptation [66]. This may have led to superior varieties that replaced preexisting domesticated populations across the Americas [67]. As genome-scale investigation of domesticates and crop wild relatives has expanded, researchers are increasingly identifying examples of adaptive introgression from wild relatives contributing to local adaptation in crops as diverse as barley [68] and date palms [69]. In some cases, even the adaptive locus itself can be identified [62,67,70]. Indeed, traditional farmers across the globe will often tolerate wild relatives in or near their fields, sometimes actively encouraging hybridization with the crop [71], with the idea that such introgression makes their crop “stronger” [72]. Perhaps the best example of this is tomato, where early farmers and breeders have brought in a host of traits from wild relatives including disease resistance [73].

If introgression from crop wild relatives generally increases maladaptation due to linkage drag and deleterious alleles, why have these processes not prevented historical gene flow? In fact, it is likely that hybridization with crop wild relatives was constrained by maladaptation. For example, ongoing gene flow between traditional varieties of maize and one of its wild relatives is depleted around loci important for maize domestication [74]. But this constraint varies across the genome; in some genomic regions, alleles from crop wild relatives may mitigate genetic load inadvertently fixed during domestication [53,75–78]. More importantly, much of the introgression between crop wild relatives and traditional varieties occurred many generations in the past and involved traditional variety populations much larger than modern breeding pools. Combined, these factors maximize the effect of recombination in breaking up linkage between beneficial alleles and maladaptive alleles at linked sites; for example, recent characterization of introgression from a wild relative in maize found the majority of introgressed segments to be quite small, often including only a single gene [67]. Large population sizes and long time periods also mean that selection by farmers—both intentional and unintentional—has had considerable opportunity to remove introgressed haplotypes with maladaptive alleles.

### Future prospects for crop wild relatives in germplasm improvement

How can wild relatives and existing germplasm resources be best used to adapt crops to the novel environments and agronomic practices that will accompany changing climates? One approach that has garnered much public attention is the use of novel genome editing techniques to “domesticate” wild plants or introduce alleles from crop wild relatives into modern germplasm. In one recent study, researchers edited a number of key genes to dramatically change the architecture and agronomic suitability of a wild relative of tomatillo [79]. In another, researchers demonstrated the feasibility and potential yield gain of introducing an allele identified in a wild relative into elite hybrid maize germplasm [80]. By segregating edits away from the initial transgenes, these approaches could circumvent regulations and concerns about genetically modified organisms. Genome editing also avoids the potential for linkage drag of deleterious alleles from crop wild relatives linked to the locus of interest.

However, we would argue that such approaches are not likely to be the most fruitful avenue for using wild relative diversity to improve crops. Domestication invariably involves changes at hundreds or thousands of alleles [56], such that it is unlikely that a crop wild relative “domesticated” via genome editing will be comparable in yield or other characteristics to modern cultivars [81]. Genetic engineering approaches also suffer from a number of logistic and scientific disadvantages [48]. First, such approaches require sufficient a priori genetic knowledge to identify the causative allele. Although causative alleles have been identified for a handful of traits in some species, the vast majority of functionally relevant diversity in most species remains entirely uncharacterized. Second, not all species are amenable to tissue culture or transformation, and within many taxa not all individuals are amenable to these practices. In maize, for example, while some private sector companies have been able to edit many varieties, public breeding and research efforts are still mostly restricted to using a small number of older inbred plants that can be readily transformed but are considered genetically inferior. This limitation means novel edited alleles still need to be backcrossed into the relevant germplasm, which carries the risk of linkage drag. Third, it is often unclear how novel edits or transgenes will behave in a new genetic background, potentially leading to undesirable epistatic interactions. Given these challenges, as well as the time, cost, and effort, only alleles with large genetic effects are generally considered for editing.

While genome editing is undoubtedly a useful tool, we argue that an effective and efficient avenue for incorporating crop wild relative diversity into modern germplasm is to use wild relative alleles already present in traditional varieties housed in germplasm banks. Germplasm repositories maintain a wealth of historical and modern genetic diversity. They increasingly include additional genetic and phenotypic data that can be used a priori to help narrow down useful material [82–84]. Increasing evidence of gene flow between traditional varieties and wild relatives during crop evolution means that germplasm collections of traditional varieties likely harbor a wealth of untapped diversity from wild relatives. Importantly, these alleles have already been filtered by the combined action of recombination and both intentional and unintentional selection (Fig 2). Crop wild relative alleles surviving in traditional varieties at appreciable frequency are thus unlikely to be linked to strongly deleterious variation and are likely to work reasonably well in a domesticated genetic background. Because these alleles are already present in a traditional variety, evaluation and later introgression into elite material is substantially easier than working directly with the wild relative. Finally, use of such materials circumvents the need to know causal alleles or mechanisms; coupled with evaluation or genomic selection, crossing with traditional varieties can effectively introgress many alleles at unknown loci across the genome without the need to understand precise causal mechanisms [85].

The incorporation of crop wild relative and traditional variety alleles into elite breeding programs is dependent on a number of factors, including the complexity of the trait or traits of interest, the ease of intercrossing, generation time, and the timeline for trait development. At one extreme, some crops may require lengthy pre-breeding interventions involving multiple crosses to bridge between diverse germplasm and relevant modern cultivars. Such bridging crosses limit the potential for genetic exchange and slow the process of introgression. But in species with genetic compatibility among wild relatives or traditional varieties, the development of multiparent populations can accelerate the identification of loci contributing to trait variation, facilitate recombination, and uncover multiple alleles at a locus that contribute to a trait [86]. In some crops, trait introgression efforts could also benefit from so-called “speed breeding” approaches, where day-night cycles, temperatures, and timing of seed harvest are manipulated to dramatically reduce the time required to grow out each generation of breeding lines [87]. When combined with marker-assisted backcrossing or genomic prediction and selection, traits of interest from a traditional variety donor can thus be rapidly introgressed while minimizing the genome-wide contribution of the donor.

For many perennial crops, including trees and clonally propagated species, conservation of wild relatives and traditional varieties can be complicated by the limited potential for reproduction by seed or the need to preserve particular strains. Species such as apple, citrus, grape, and avocado are preserved in living nurseries, where many plants may consist of distinct genetics aboveground grafted to rootstocks that are tolerant of local growing conditions and soil pests [88]. While these collections may occupy large tracts of land with “permanent” plantings, they also offer some advantages. These include the opportunity to observe and harvest fruits grown under a variety of weather conditions over many growing seasons.

### Safeguarding diversity for the future

We are losing genetic resources from farmers’ fields through the replacement of traditional varieties by modern cultivars [89] and from the wild in part due to the very same global challenges that their use could contribute to solving: climate and environmental changes [90,91]. Conserving and making available crop genetic resources for current and future use in breeding, research and cultivation is the core mandate of genebanks all around the world [92]. While global germplasm collections are far from complete, and germplasm samples of traditional varieties and natural populations of crop wild relatives are particularly under-collected and under-conserved [93,94], they remain a critical—and in some cases our only—source of wild relative variation. *Triticum tiompheevii*, for example, is a wild relative of wheat that is most likely extinct in situ but still available from ex situ collections [95].

Germplasm repositories are increasingly pushing to make their resources available. They do so most effectively by sharing germplasm under agreed terms as described under the multilateral system of the International Plant Treaty [96] or similar arrangements that are set up to encourage use by private and public sector users. Similarly, large-scale, systematic initiatives are taking place to evaluate and assess traits of interest in germplasm samples [82,84,97,98], providing key data that is useful for initiating pre-breeding programs. Taking advantage of these resources, including traditional variety germplasm and the diverse wild relative alleles they contain, may well prove key in adapting crops to rapidly changing global environments.
